# Spatio-temporal variation of skeletal Mg-calcite in Antarctic marine calcifiers

**DOI:** 10.1371/journal.pone.0210231

**Published:** 2019-05-07

**Authors:** Blanca Figuerola, Damian B. Gore, Glenn Johnstone, Jonathan S. Stark

**Affiliations:** 1 Smithsonian Tropical Research Institute (STRI), Panama City, Panama; 2 Biodiversity Research Institute (IrBIO), University of Barcelona, Barcelona, Catalonia, Spain; 3 Department of Environmental Sciences, Faculty of Science and Engineering, Macquarie University, Sydney, Australia; 4 Antarctic Conservation and Management Program, Australian Antarctic Division, Hobart, Tasmania, Australia; University of Barcelona, SPAIN

## Abstract

Human driven changes such as increases in oceanic CO_2_, global warming, petroleum hydrocarbons and heavy metals may negatively affect the ability of marine calcifiers to build their skeletons/shells, especially in polar regions. We examine spatio-temporal variability of skeletal Mg-calcite in shallow water Antarctic marine invertebrates using bryozoan and spirorbids as models in a recruitment experiment of settlement tiles in East Antarctica. Mineralogies were determined for 754 specimens belonging to six bryozoan species (four cheilostome and two cyclostome species) and two spirorbid species from around Casey Station. Intra- and interspecific variability in wt% MgCO_3_ in calcite among most species was the largest source of variation overall. Therefore, the skeletal Mg-calcite in these taxa seem to be mainly biologically controlled. However, significant spatial variability was also found in wt% MgCO_3_ in calcite, possibly reflecting local environment variation from sources such as freshwater input and contaminated sediments. Species with high-Mg calcite skeletons (e.g. *Beania erecta*) could be particularly sensitive to multiple stressors under predictions for near-future global ocean chemistry changes such as increasing temperature, ocean acidification and pollution.

## Introduction

Human stressors such as contaminants and increases in oceanic CO_2_ will lead to significant changes in global ocean chemistry in the near future. Some of the expected consequences of the increase in oceanic CO_2_ are a reduction in seawater pH of 0.3–0.5 pH units by 2100, leading to changes to the carbonate system including a decrease in the carbonate saturation state (Ω) [1] and changes to metal speciation, availability and toxicity in seawater [2]. These human driven changes might negatively affect the ability of marine calcifiers to build their calcified skeletons/shells. There is a need therefore to understand the basic underlying properties of organisms hypothesised to be vulnerable to such changes, for example to understand calcium carbonate mineralogy. Marine calcifiers produce a variety of mineralogical forms (polymorphs) including aragonite, calcite and calcite minerals containing a range of magnesium (Mg) content. In particular, seawater in polar regions is acidifying at a faster rate than elsewhere [3] and organisms at high latitudes may also be more susceptible to contamination (e.g. trace metals and Persistent Organic Pollutants (POPs)) compared to other regions due to their slower metabolism, growth and larval development and consequent slower detoxification processes, slower colonisation rates and lower deposition of wt% MgCO_3_ in calcite [4–8]. Synergistic effects of anthropogenic driven environmental change could mean a significant degradation or even loss of calcareous reef habitats and sediment deposits used as shelter, substrate and food for many benthic communities [5,9,10].

Seawater carbonate saturation state, pH and temperature play an important role in the incorporation of Mg into calcified structures, however, other environmental (e.g. salinity and Mg/Ca ratio in seawater) and biological (e.g. species-specific mineralogies and skeletal growth rate) factors also mediate this process [11–13]. Therefore, the extent of spatial and temporal variations in skeletal Mg-calcite, especially organisms inhabiting Antarctic waters, remains unclear.

Laboratory studies are useful in demonstrating the effects of environmental stressors in controlled conditions, for example that heavy metal toxicity may reduce growth rates of calcifying marine invertebrates [14,15]. However, experiments in the field can also contribute to our understanding of how these effects will manifest on calcified organisms living in complex ecosystems [16]. Such studies allow simultaneous exploration of different physical, biological and anthropogenic factors (e.g. depth, species interactions and pollution) which likely interact in their resulting effects on organisms. They also allow the estimation of the importance of spatial and temporal variation in response variables (such as calcification and mineralogy), which are essential to understanding relative risks or vulnerabilities of species to climate change effects. Without an understanding of environmental variation in habitats predicted to experience environmental change, along with an understanding of natural variation in species responses, it is not possible to make predictions about how species may respond to such changes. Predicted environmental changes may well be within the boundaries of what species experience or may push them to limits of physiological tolerances.

Ecosystem engineers such as calcifying bryozoans and spirorbid polychaetes contribute to habitat structure and resilience, especially in Antarctica where these taxa are widespread and diverse [17–19]. They play important roles in creating reef frameworks and habitat complexity that enhance biodiversity and can prevent storm damage [20,21]. Spirorbid polychaetes and bryozoans are amongst the most prominent early colonizers in Antarctica [7] but the CaCO_3_ biomineralization of their calcareous tube is relatively unknown [7,22,23]. This study investigates the spatio-temporal variability in skeletal Mg-calcite in calcifying Antarctic bryozoans and spirorbid polychaetes [22,24–26]. Samples were collected from settlement panels deployed around Casey Station in East Antarctica including from undisturbed and human-impacted areas. The objectives were: (1) to test whether there is spatial variability in skeletal Mg-calcite at a range of spatial scales around Casey and at different water depths; (2) to test whether there is temporal variability (between settlement panels at 3 and 9 y) in skeletal Mg-calcite and (3) to test whether or not there are differences in mineralogy between impacted areas (sediments contaminated with petroleum hydrocarbons and heavy metals) and non-impacted areas around Casey Station.

## Materials and methods

### Study area

**Fig 1:**
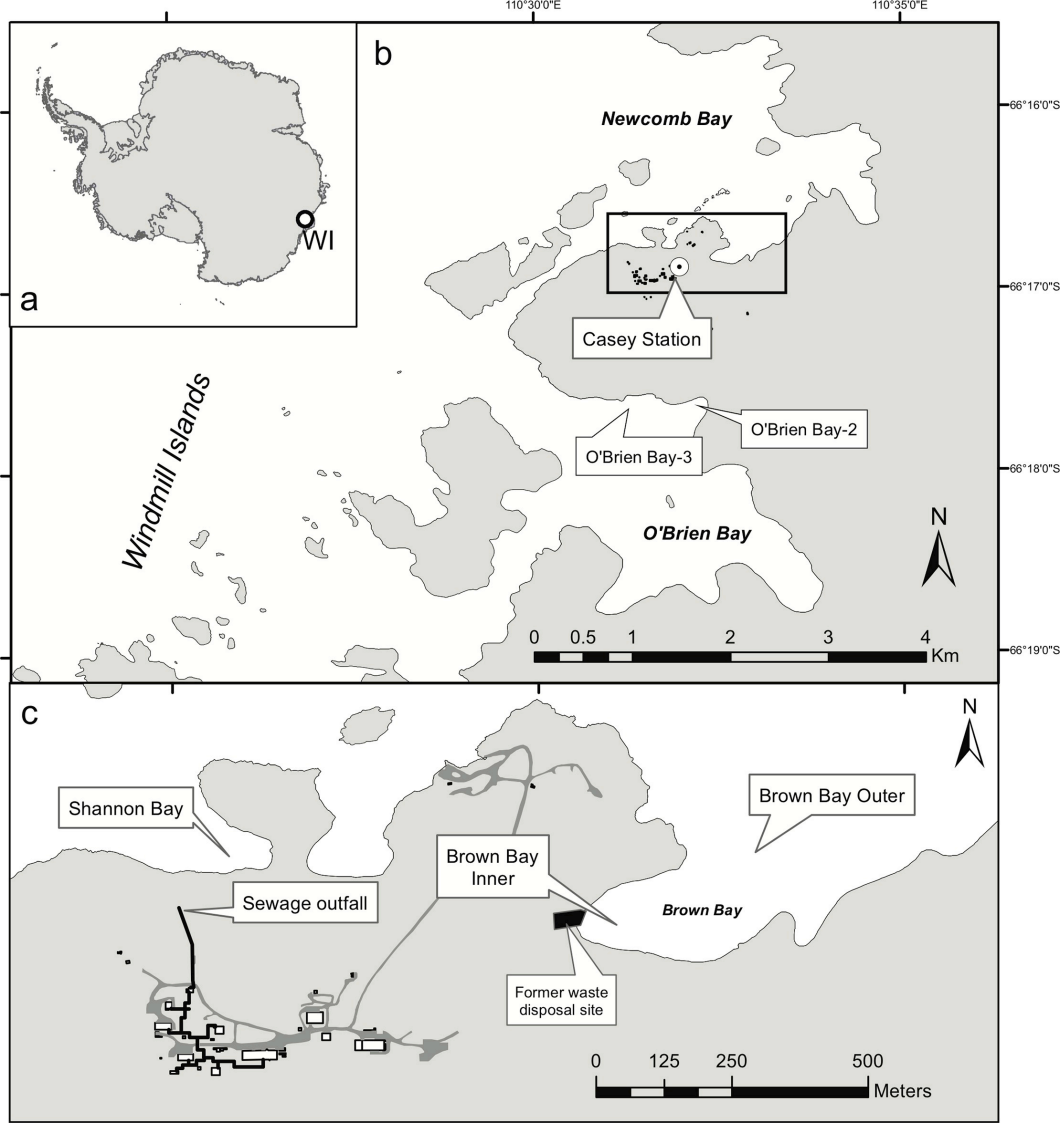
Location of study site. a) Location of Windmill Islands (WI) in East Antarctica; b) the Windmill Islands area around Newcomb and O’Brien Bays showing the position of Casey station and sites in O’Brien Bay with inset c) Casey station, and Shannon and Brown Bays. The topographic data was obtained from the Australian Antarctic Data Centre (https://data.aad.gov.au/aadc/portal/drill_down.cfm?id=14) and used with permission under the Creative Commons Attribution 3.0 Unported License. The map was made with ArcGIS 10.4.

The study area is located around Casey Station (66° 17' S, 110° 32' E) in the Windmill Islands, on the coast of Wilkes Land, East Antarctica (Fig 1). The shallow marine benthic environment is a heterogeneous mosaic of sediments ranging from mud to sand, gravel, cobbles, boulders and bedrock [27]. The experiment was conducted at five locations: Brown Bay outer and Brown Bay inner, O'Brien Bay-1 and O’Brien Bay-2 and Shannon Bay. These bays are covered by sea ice (1.2−2.0 m thick) for most of the year and are ice free for 1−2 months of the year in Brown Bay and 3−4 months in the other bays. Sea ice reduces physical disturbance of the seabed by icebergs and from wave or wind induced turbulence. Access to these sites was permitted by the Australian Antarctic Division, and the work did not involve endangered or protected species

Brown Bay is in the southwestern corner of Newcomb Bay. The southern shore is composed of ice cliffs and its western and northern shores are an ice bank at the end of Thala Valley, the location of a former waste disposal site [28]. During summer, a melt stream flows down Thala Valley into Brown Bay. O'Brien Bay is on the southern side of Bailey Peninsula and the shore consists of ice cliffs, 2−30 m high [27]. Shannon Bay is bordered by ice cliffs 2−15 m high and has a wastewater outfall discharging into the ice cliff adjacent to the bay. Brown Bay and Shannon Bay are adjacent to sources of contamination (~50 m from the waste disposal site and ~100 m from the sewage outfall, respectively) and are contaminated with petroleum hydrocarbons and heavy metals which are much higher in comparison to the control sites (Table 1) [29]. Salinity and temperature were measured in previous studies and all sites had similar oceanographic conditions, with small seasonal fluctuations in salinity (33–36‰) and temperature (-0.5 to -1.9°C) [30,31].

**Table 1:** Metal and total petroleum hydrocarbon (TPH) levels in marine sediments at each site. Standard error (SE) in brackets, n = number of replicates.

Brown Bay Inner	1997–98	7	18.62	(4.30)	15.59	(4.24)	2718.76	(845.43)	48.77	(11.18)	0.11	(0.02)	6.54	(1.73)	36.52	(8.73)	153.9374	(37.91)
Brown Bay Inner	1998–99	30	14.68	(1.88)	21.32	(3.04)	3676.47	(519.44)	69.81	(8.27)	0.15	(0.01)	11.76	(1.72)	49.1765	(5.28)	425.3333	(65.33)
Brown Bay Inner	2005–06	8	16.21	(2.16)	29.47	(4.58)	4541.90	(753.00)	68.68	(9.77)	0.15	(0.01)	14.22	(3.35)	41.81	(3.72)		
Brown Bay Inner	2006–07	8	9.74	(2.56)	14.35	(2.68)	2192.59	(406.38)	54.56	(9.91)	0.15	(0.03)	7.97	(1.72)	29.03	(3.85)		
Shannon Bay	1997–98	8	21.96	(3.99)	2.30	(0.39)	287.24	(76.62)	1.80	(0.19)	0.03	(0.00)	0.37	(0.09)	15.30	(0.92)	76.1216	(20.28)
Shannon Bay	2006–07	24	9.87	(1.74)	6.50	(1.72)	472.24	(29.98)	2.10	(0.23)	0.02	(0.00)	0.98	(0.28)	13.18	(1.30)		
Brown Bay Outer	1997–98	4															23.03734	(9.20)
Brown Bay Outer	1998–99	12	18.47	(5.44)	4.60	(0.67)	463.73	(119.69)	8.62	(2.90)	0.03	(0.00)	1.13	(0.38)	20.92	(2.76)	235	(103.44)
O'Brien Bay-2	1997–98	8	9.72	(1.30)	0.84	(0.25)	145.54	(20.83)	0.50	(0.05)	0.02	(0.00)	0.02	(0.00)	10.24	(1.92)	8.432372	(3.14)
O'Brien Bay-2	1998–99	8	8.63	(1.61)	0.58	(0.07)	163.09	(20.80)	0.42	(0.05)	0.02	(0.00)	0.02	(0.00)	6.16	(0.58)		
O'Brien Bay-2	2006–07	8	7.41	(0.75)	1.04	(0.16)	374.78	(20.84)	0.56	(0.04)	0.02	(0.00)	0.01	(0.00)	9.42	(0.51)		
O'Brien Bay-3	1997–98	8	6.61	(0.53)	0.47	(0.06)	74.24	(6.93)	0.23	(0.02)	0.02	(0.00)	0.02	(0.00)	6.26	(0.52)	30.2556	(14.34)

### Sampling design

**Fig 2:**
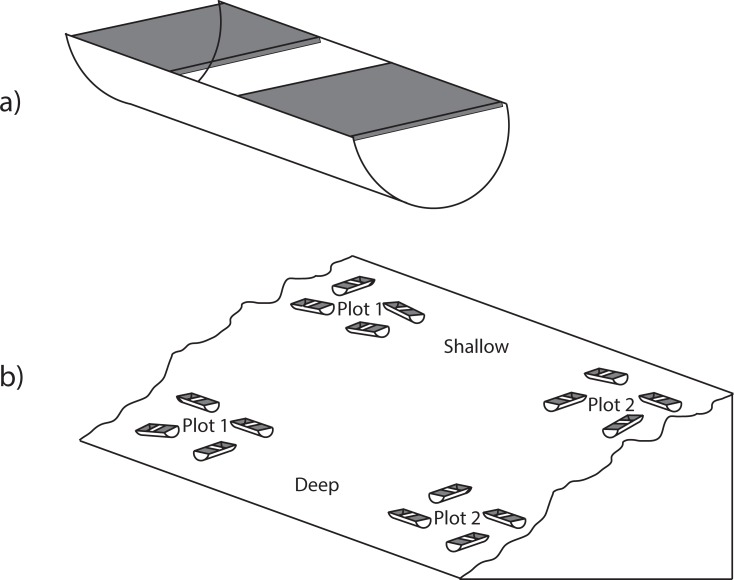
Settlement panels. (a) Two tiles were situated in the top of a trough formed from one-half of a PVC pipe; (b) Experimental design at each site showing groups of eight tiles, two shallow sites and two deep sites.

The settlement panels consisted of unglazed porcelain tiles (15 × 15 cm), with a slightly textured upper surface and 3 × 3 cm slightly raised grid (~1 mm high) on the lower surface (Fig 2A). Two tiles were attached 10 cm apart to the top edge of a trough made from one-half of a 40 cm long, 15 cm diameter PVC stormwater pipe (see the detailed experimental design in [7]). Tiles were deployed and collected by divers at 5 sites (Table 2). Three sites are known to be impacted by human activities: Brown Bay Inner (BBI) and Brown Bay Outer (BBO), ~50 m and 500 m from an abandoned waste disposal site respectively, and Shannon Bay (SB), ~100 m from a sewage outfall)(Fig 1), and have contaminated sediments (Table 1) and disturbed benthic communities [29,32]. There were two uncontaminated control sites: O'Brien Bay-2 (OB2) and O’Brien Bay-3 (OB3) in O’Brien Bay, south of Casey Station (Fig 1C). Tiles were deployed at two depths at each site (6 to 10 m and 19 to 22 m) except at BBI and BBO. Colonization and assemblage development at species level are highly site-specific and depth-dependent in Antarctica [33]. At each depth, tiles were deployed in two plots ~20 m apart in groups of eight tiles (four troughs ~1 to 2 m apart), with a total of 32 tiles at each location (Fig 2B). It was not possible to deploy deep tiles at BBI, as there is no deep habitat near the waste disposal site, nor was it possible to deploy shallow tiles at BBO. However, at these two sites, two groups of eight tiles (in plots of four troughs) were placed ~50 m apart to examine small-scale spatial variation. Tiles were deployed between 15 November and 31 December 1997 and collected after 3 y (in December 2001) and 9 y (in November 2006). One tile was collected from each of two randomly selected troughs at each plot/depth at each sampling time (a total of eight per location).

**Table 2:** Depth and coordinates of the sites.

Sites	Latitude	Longitude	Depth (m)
Brown Bay Inner	-66.28	110.541	6
Brown Bay Outer	-66.28	110.547	19
Shannon Bay Shallow	-66.28	110.523	6
Shannon Bay Deep	-66.28	110.523	20 to 22
O'Brien Bay-2 Shallow	66.294	110.537	8 to 10
O'Brien Bay-2 Deep	66.294	110.537	20 to 22
O'Brien Bay-3 Shallow	-66.29	110.52	6 to 7
O'Brien Bay-3 Deep	-66.29	110.52	20 to 22

### Collection and identification

**Fig 3:**
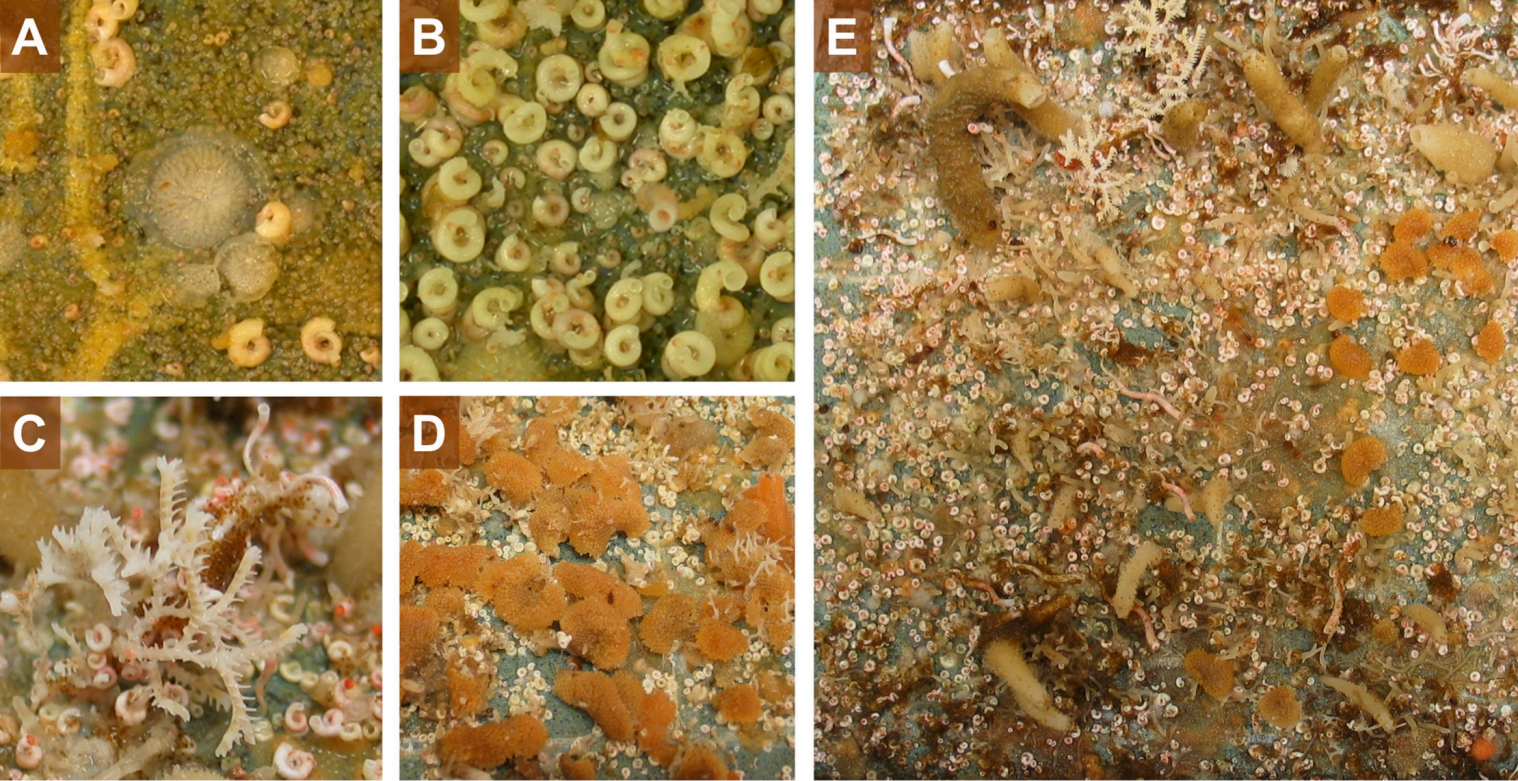
**Images of the tiles recovered after 6 (A-B) and 9 (C-E) years.** A) A cyclostome colony of *Disporella* sp.; B) Spirorbid species which form closely coiled tubes (referred here as *Spirorbis* sp.1); C) a cyclostome colony of *Idmidronea* sp. and the spirorbid species which form tusk-shaped tubes (top right; *Spirorbis* sp. 2); D) colonies of the cheilostome *Beania erecta*. E) entire tile colonized by bryozoan colonies, spirorbid polychaetes and different species of sponges.

Divers collected the tiles which were then preserved in 95% ethanol (Fig 3). Bryozoan colonies were identified to species level in the laboratory using an optical microscope and guide [34]. In subtidal benthic communities, spirorbid polychaetes and bryozoans are characteristic early colonists and dominant species until they are outcompeted by other taxa, particularly by sponges and ascidians (Clarke 1996). Six abundant and widely distributed Antarctic bryozoan species selected for this study were the cheilostomes *Arachnopusia decipiens* Hayward & Thorpe, 1988, *Inversiula nutrix* Jullien, 1888, *Beania erecta* Waters, 1904, *Ellisina antarctica* Hastings, 1945, and the cyclostomes *Disporella* sp. and *Idmidronea* sp. Two abundant spirorbid polychaete species of the genus *Spirorbis* were also chosen: one morpho-species which forms closely coiled tubes (*Spirorbis* sp.1) and another morpho-species with tusk-shaped tubes (*Spirorbis* sp. 2).

### Mineralogical analyses

Wherever possible, three specimens of each species from each location, each depth and each time were selected for mineralogy analyses. Samples were dissected from 54 tiles with a scalpel blade and a piece (2 mm^2^) from the growing edge of the bryozoan colonies and entire spirorbid polychaete tubes were removed for analysis. After being air-dried, epibionts were removed to avoid mineralogical contamination.

Samples were powdered using an agate mortar and pestle and placed on a Si-crystal low background holder for analysis. X-ray diffractograms were collected from 5 to 90° 2θ with a Panalytical X'Pert Pro MPD diffractometer, using tube conditions of 45 kV, 40 mA, CuK_α_ radiation, X'Celerator detector, Bragg Brentano geometry, and a slew rate of 5° 2θ per minute. Diffractometer performance was constrained by measurement of silver behenate and a single silicon crystal. Limits of detection vary from 0.1 to ~2 wt% depending on the crystallinity of the phase. Identification of minerals, including poorly ordered phases, was conducted using Panalytical's Highscore Plus software v2.2.4, with ICDD PDF2 and PAN-ICSD databases. Mg-Ca substitution in calcite was quantified via linear interpolation of the angle of the 104 reflection from calcite (CaCO_3_) (0% Mg-calcite at XX° 2θ) to magnesite (MgCO_3_) (100% Mg-calcite at YY° 2θ) [35].

**Fig 4:**
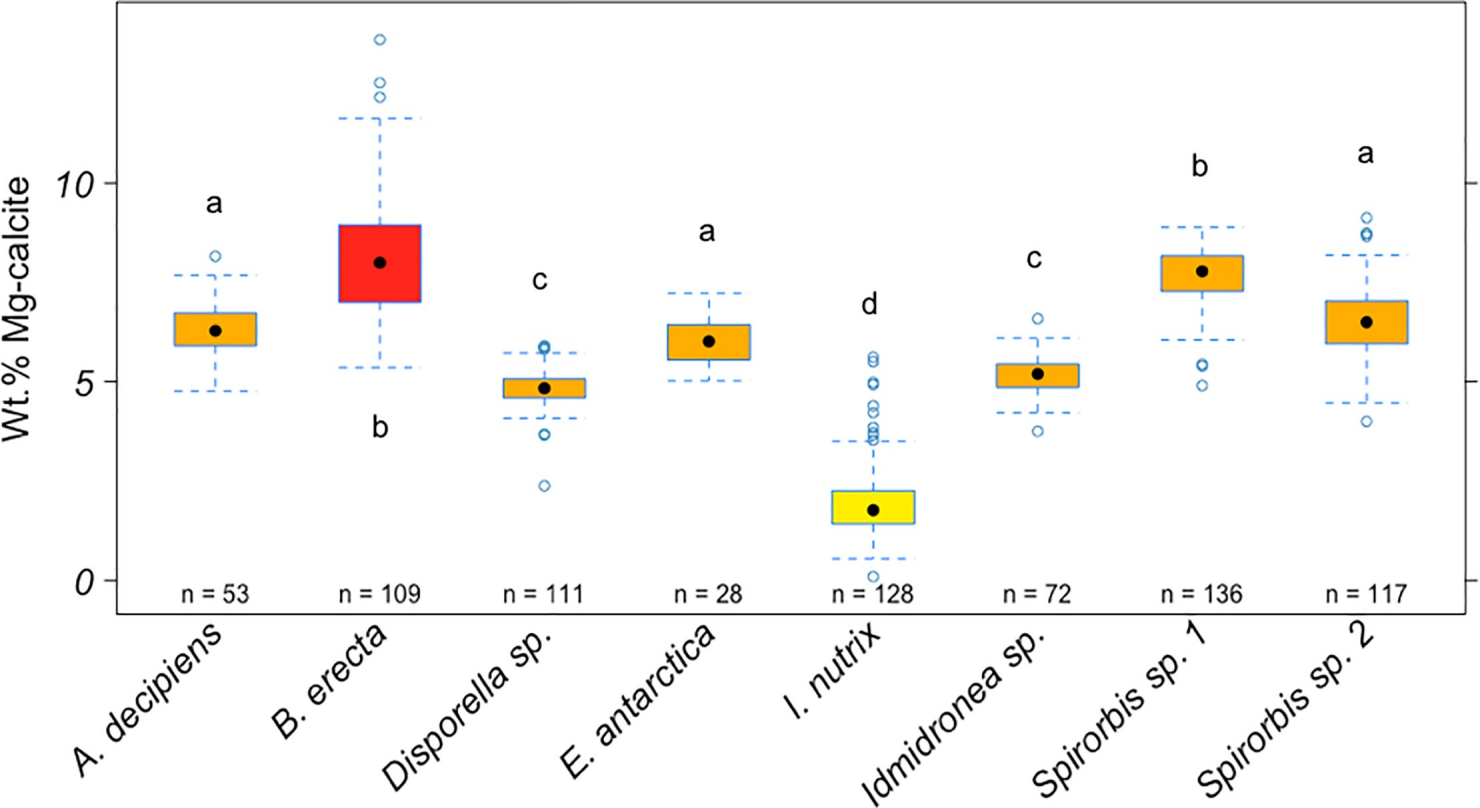
Mean values (±standard error) of wt% MgCO_3_ in calcite in the eight Antarctic species. Boxes depict standard deviation around mean (mid-line); tail indicates range. Colours indicate high-Mg calcite (red), intermediate-Mg calcite (orange) and low-Mg (yellow). Letters indicate results of PERMANOVA tests and show significant differences between species, as indicated by different letters. Species that share the same letter are not significantly different.

**Fig 5:**
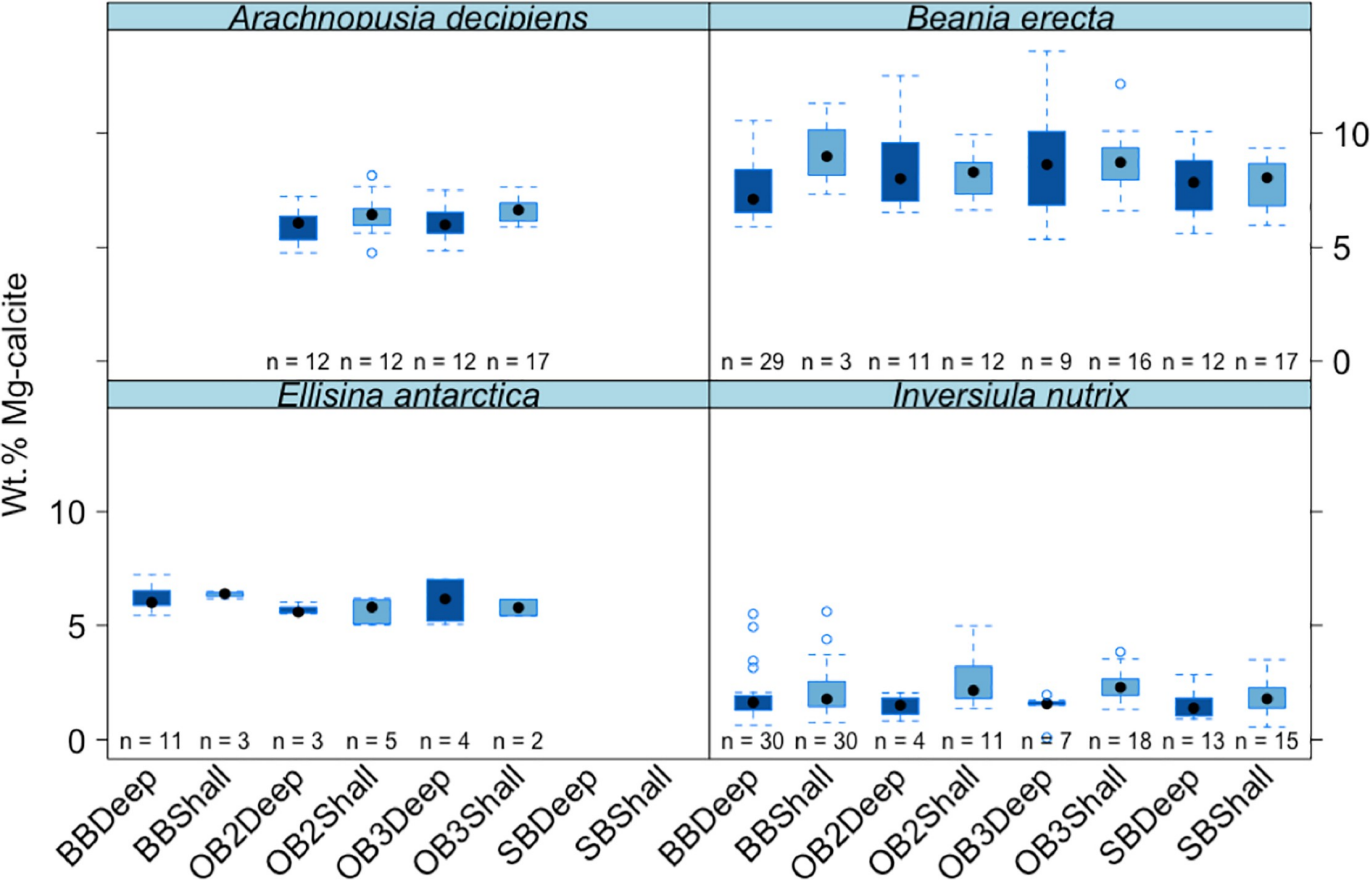
Mean values (±standard error) of wt% MgCO_3_ in calcite in the four Antarctic cheilostome species among different sites and depths. Boxes depict standard deviation around mean (mid-line); tail indicates range. Colours indicate deep and shallow waters.*Brown Bay = BB, *OB2 = O'Brien Bay -2*, *OB3 = O’Brien Bay-3*, *SB = Shannon Bay*. *Deep = Deep*, *shallow = Shall*.

**Fig 6:**
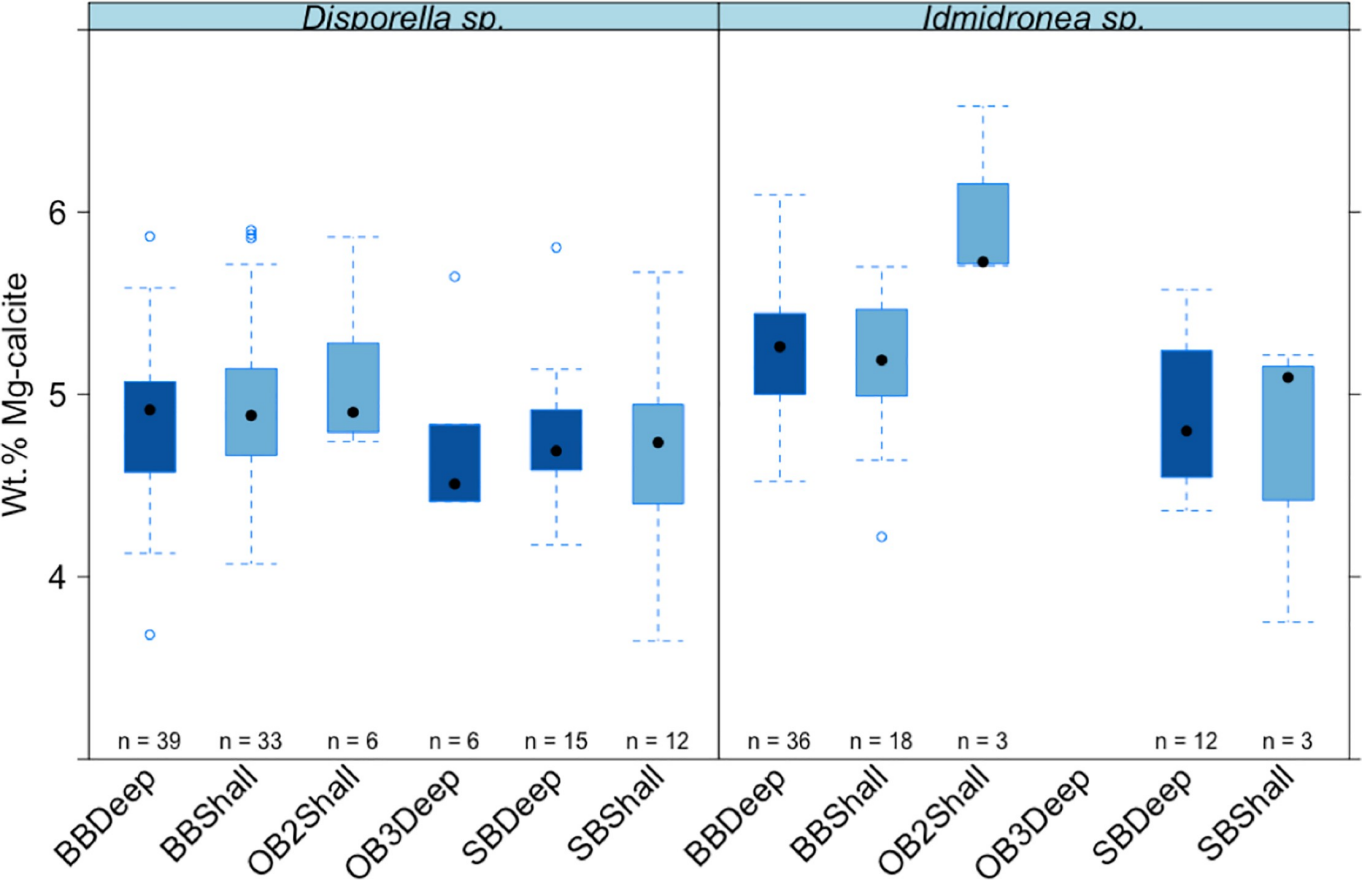
Mean values (±standard error) of wt% MgCO_3_ in calcite in the two Antarctic cyclostome species among different sites and depths. Boxes depict standard deviation around mean (mid-line); tail indicates range. Colours indicate deep and shallow waters. *Brown Bay = BB, *OB2 = O'Brien Bay -2*, *OB3 = O’Brien Bay-3*, *SB = Shannon Bay*. *Deep = Deep*, *shallow = Shall*.

**Fig 7:**
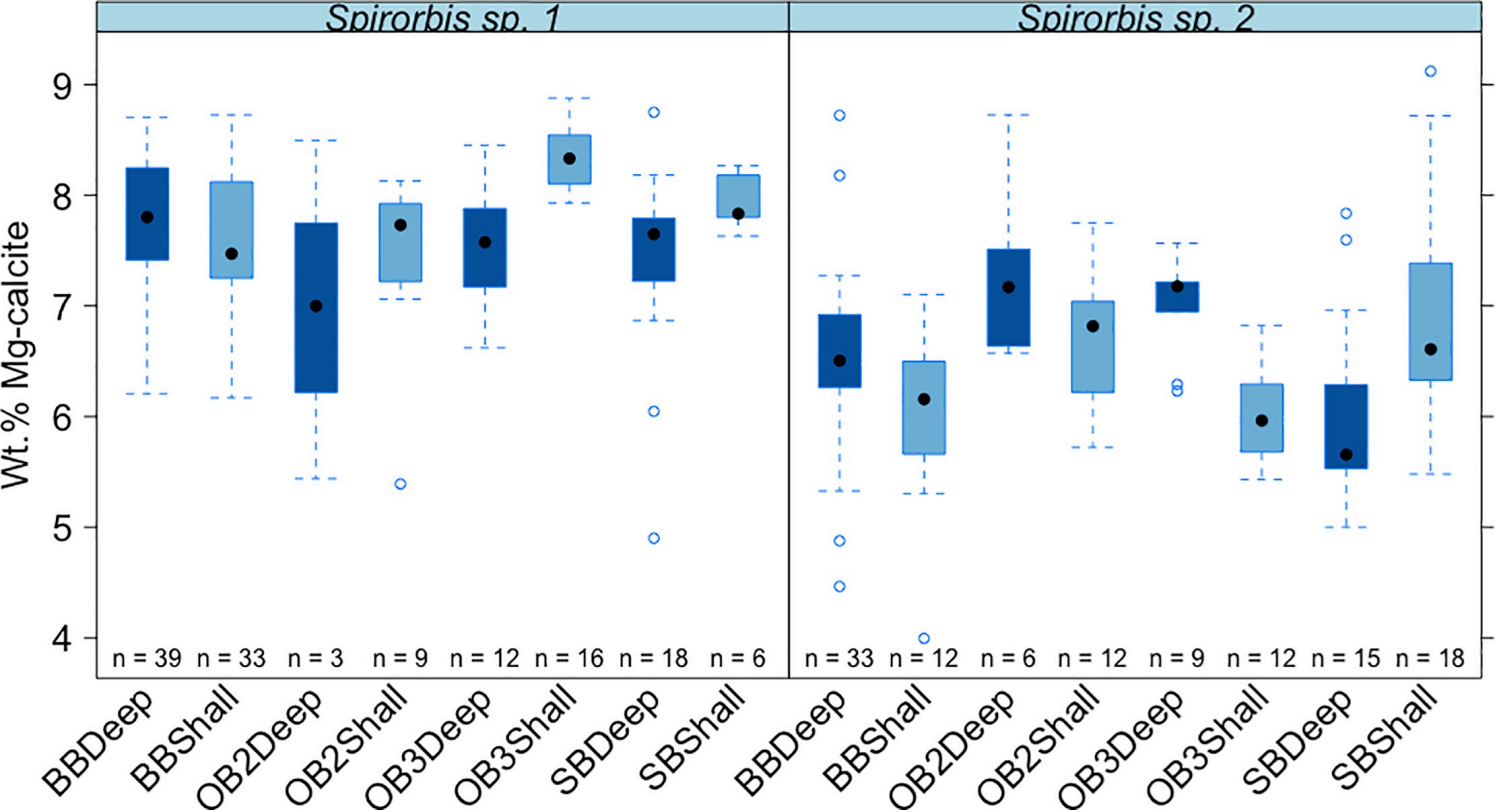
Mean values (±standard error) of wt% MgCO_3_ in calcite in the two Antarctic spirorbid species among different sites and depths. Boxes depict standard deviation around mean (mid-line); tail indicates range. Colours indicate deep and shallow waters. *Brown Bay = BB, *OB2 = O'Brien Bay -2*, *OB3 = O’Brien Bay-3*, *SB = Shannon Bay*. *Deep = Deep*, *shallow = Shall*.

Mineralogies were determined for 754 specimens belonging to six bryozoan species (four cheilostome and two cyclostome species) and two spirorbid polychaete species (Figs 4–7). All species were entirely calcitic. Skeletons were divided into low-Mg calcite (LMC; 0–4 wt% MgCO_3_), intermediate-Mg calcite (IMC; 4–8 wt% MgCO_3_) and high-Mg calcite (HMC; >8 wt% MgCO_3_) categories, following a common classification [36].

### Chemical analyses

Sampling and analysis of metals in sediments were done as described in Stark et al. [29,37]. Briefly, samples for heavy metal analysis were sieved through a 2mm mesh screen with clean 0.45μm filtered seawater to remove coarse material. The <2mm fraction was oven dried at 103°C and dried samples were broken with a spatula and 5g was digested in 1M HCl for 4h on a shaker plate as recommended by Scouller et al. [38] and Snape et al. [39]. Extracts were filtered through a 0.45 μm cellulose nitrate filter and analysed for a range of metals by Quadrupole-ICPMS (Perkin Elmer Elan 6100-DRC). All glass and plasticware used in the processing were washed in 10% HNO_3_ and triple rinsed in MQ+ deionised water. Metal concentrations in procedural blanks were typically three orders of magnitude lower than in control sediments. Sampling of sediments for hydrocarbons was done as described in Stark et al. [29] and analysis of total petroleum hydrocarbons was done as described in Stark et al. [40].

### Statistical analyses

To test for differences among species, year, depth and site, a 4 factor PERMANOVA [41–44] was used. The design included the fixed factors (species, year and depth) and site nested in the combination of species, year and depth. Where significant differences were found, post hoc pairwise PERMANOVA tests were performed. PERMANOVA was done on untransformed skeletal Mg-calcite using a Euclidean distance similarity matrix, which is directly analogous to a univariate ANOVA [45] but is well suited to unbalanced designs, such as is the case in this study (e.g. for depth). To test for differences in mineralogy between contaminated and control sites a three factor design was used with Impact (BBI, BBO and SB vs OB), Species and Site (nested in impact x species). The program PRIMER7 (V7.0.13, PRIMER-e, Quest Research Limited) was used for these analyses and the graphical displays were produced using R version 3.5.0 [46]. The package Lattice [47] was called to perform some of the analysis.

## Results

Statistical analysis of mineralogy was done in a four factor design with Species, Year, Depth and Site (nested in species, year and depth) and indicated significant effects of species, depth and site but not year (Table 3). There was a strong significant difference in the mean wt% MgCO_3_ in calcite among species and sites (Fig 4–7; Table 3) and there was a small but significant difference among depths (Table 3). There were no significant interactions between species, year or depth (Table 3), indicating differences between species were consistent. There was no effect of year of sampling.

**Table 3:** PERMANOVA testing for the effects of species, year and depth on the Mg content in calcite. The design included the fixed factors (species, year and depth) and site nested in the combination of species, year and depth. PERMANOVA was done on untransformed skeletal Mg-calcite using a Euclidean distance similarity matrix. % var = Estimates of components of variation of Mg content in calcite for each factor in the analysis. Bold type indicates significant difference at p = 0.001.

*Source*	*Df*	*SS*	*MS*	*Pseudo- F*	*P*	*% var*
**Species**	**7**	**2140.1**	**305.73**	**226.9**	**0.001**	62.43
Year	1	0.84573	0.84573	0.66618	0.412	0
**Depth**	**1**	**6.4576**	**6.4576**	**5.0867**	**0.029**	4.65
SpxYe	7	3.8223	0.54604	0.40524	0.888	0
Spxde	7	8.3086	1.1869	0.88088	0.532	0
YexDe	1	0.26294	0.26294	0.20712	0.632	0
SpxYexDe	7	8.1498	1.1643	0.86405	0.513	0
**Site (SpxYexDe)**	**53**	**79.796**	**1.5056**	**2.0489**	**0.001**	8.87
Residual	669	491.6	0.73483			24.05
Total	753	3725.3				

### Intra- and interspecific variation in skeletal Mg-calcite

*Arachnopusia decipiens* (n = 53), *Ellisina antarctica* (n = 28), *Idmidronea* sp. (n = 72) and *Disporella* sp. (n = 111), *Spirorbis* sp. 1 (n = 136) and sp. 2 (n = 117) had skeletons of IMC (4–8 wt% MgCO_3_ in calcite) (Fig 4). Of the remaining species, *Beania erecta* (n = 109) consisted of HMC (>8 wt% MgCO_3_ in calcite) and *Inversiula nutrix* (n = 128), of LMC (2–4 wt% MgCO_3_ in calcite). The pairwise tests for species showed that there is a significant difference in the mean wt% MgCO_3_ in calcite among most species except *E*. *antarctica* versus *Spirorbis* sp. 2 (PERMANOVA pairwise test: *P* = 0.067) and *A*. *decipiens* (*P* = 0.051), *Spirorbis* sp. 2 versus *A*. *decipiens* (*P* = 0.402), *Spirorbis* sp. 1 versus *B*. *erecta* (*P* = 0.154), *Idmidronea* sp. versus *Disporella* sp. (*P* = 0.051) (Fig 4; Table 4). Differences in skeletal Mg-calcite between species contributed to >62% of the total observed variation (Fig 4; Table 4). Variation within species (residual variation among replicates within same depth, year and site) contributed ~24% to overall variation.

**Table 4:** Post hoc pairwise permutational multivariate analysis of variance (PERMANOVA) tests for differences in Mg content among species. PERMANOVA was done on untransformed skeletal Mg-calcite using a Euclidean distance similarity matrix. Values from t tests shown, with bold type indicates significant difference at p = 0.001, except where *p<0.01.

	*I*. *nutrix*	*A*. *decipiens*	*Disporella* sp.	*Idmidronea* sp.	*B*. *erecta*	*Spirorbis* sp. 1	*Spirorbis* sp. 2
*E*. *antarctica*	*17.25	2.15	**7.70**	**4.12**	**5.33**	**7.71**	1.94
*Spirorbis* sp. 2	**24.95**	0.87	**9.49**	**6.01**	**6.03**	**6.12**	
*Spirorbis* sp. 1	**35.76**	**6.76**	**19.02**	**13.34**	1.48		
*B*.*erecta*	**27.55**	**5.20**	**14.17**	**10.38**			
*Idmidronea* sp.	**17.32**	*6.03	2.23				
*Disporella* sp.	**18.66**	**10.37**					
*A*. *decipiens*	**21.29**						

### Spatio-temporal analysis in skeletal Mg-calcite

Differences among sites (which are confined to comparisons among the same species, depths and years as this was a nested factor in the analysis, i.e. site was nested in species, year and depth) were significant and contributed ~9% of the variance (Table 3, Figs 5–7).

There was a small but significant effect of depth (Table 3), with differences in skeletal Mg-calcite between different depths contributing ~5% to the estimated total variation (Figs 5–7).

The effects of contamination on mineralogy were analysed in a three factor design with Impact, Species and Site (nested in impact x species). There was no significant overall effect difference in skeletal Mg-calcite concentrations between contaminated (sediments contaminated with petroleum hydrocarbons and heavy metals; Brown Bay Inner (BBShall) and Shannon Bay (SB)) and control sites (Brown Bay Outer (BBDeep), O'Brien Bay-2 (OB2) and O’Brien Bay-3 (OB3)) (Table 5) nor any significant difference for any individual species between contaminated and control sites (species x impact interaction, Table 5).

**Table 5:** Results of PERMANOVA analysis testing for the effects of human impact on the Mg content in calcite, with tests for each species (impact x species). PERMANOVA was done on untransformed skeletal Mg-calcite using a Euclidean distance similarity matrix. % var = Estimates of components of variation of Mg content in calcite.

*Source*	*df*	*MS*	*Pseudo-F*	*P(perm)*	*% var*
Impact	1	2.66	2.78	0.114	3.9
Species	7	187.63	173.47	0.001	61.1
Impact x species	6	1.16	1.12	0.431	2.2
Site (Sp. x impact)	21	1.28	1.67	0.039	5.0
Residual	718	0.77			27.8
Total	753				

## Discussion

This study evaluates the variability of wt% MgCO_3_ in calcite in the skeletons of a large number of specimens of common bryozoan and spirorbid taxa in coastal Antarctic waters, one of the regions expected to be first affected by global change in the near future and where taxa may be more susceptible to contamination than lower latitudes. To our knowledge, this is the first study addressing the spatio-temporal variability of skeletal Mg-calcite using bryozoan and spirorbid polychaete species as models in an experiment of settlement tiles in Antarctica. These two taxonomic groups, and some genera and species studied here (e.g. *Arachnopusia*, *Beania*, *E*. *antarctica*), are common components of hard-substratum sessile assemblages across a wide range of cold latitudes (e.g. the South American Region) [48]. All species had calcitic skeletons, which is consistent with our expectations from previous research on bryozoans and spirorbids from high latitudes [26,49–52]. This trend is possibly due to low temperatures favouring the deposition of calcite over aragonite as the latter is more susceptible to dissolution in cold waters [51].

### Inter- and intraspecific variability in skeletal Mg-calcite

Inter-specific differences in the mean wt% MgCO_3_ in calcite were the largest source of variation overall. Remarkably, there were significant differences in the skeletal Mg-calcite between all bryozoan species except between *E*. *antarctica* and *A*. *decipiens* (which were on the borderline of a < 5% probability of significant difference) and between the two cyclostome species. There was a wide range of wt% MgCO_3_ in calcite measurements in the bryozoan and spirorbid polychaete species studied here, from 0.1–13.1 wt% MgCO_3_ in calcite. These values are not surprising for wt% MgCO_3_ in calcite in bryozoans, which can range from 0 to 14 [49,50], nor for spirorbids, which range from 7 to 15 [22]. Only one species was classified as LMC (*Inversiula nutrix*); five as IMC (*Arachnopusia decipiens*, *Ellisina antarctica*, *Disporella* sp., *Idmidronea* sp. and *Spirorbis* sp. 1 and sp. 2); and one as HMC (*Beania erecta*). Most cheilostome bryozoans secrete IMC [49,50], which is further confirmed by this study. To our knowledge, this study also represents the first mineralogical measurements made for the species *A*. *decipiens*. Our values are within the range of wt% MgCO_3_ in calcite obtained by previous studies in Antarctic cheilostome bryozoans except for *B*. *erecta*, which has been reported to be IMC [26,53,54]. The skeletons of *B*. *erecta* in our study showed higher mean Mg values even though the range was highly variable (5.3–13.6 wt% MgCO_3_ in calcite). This species exhibits a wide geographical distribution in Antarctica and it could explain the broad range of wt% MgCO_3_ in calcite found here. Other studies have reported ICM and LMC skeletons in *E*. *antarctica* and *I*. *nutrix*, respectively, from King George and Adelaide Islands (Antarctic Peninsula)[24,26,50]. Significant differences were found in the skeletal Mg-calcite even between cheilostomes of the same suborder Flustrina, in *B*. *erecta* and *E*. *antarctica*, although they belong to different families (Beaniidae and Ellisinidae, respectively). Coinciding with our results on the cyclostome genera, *Idmidronea* sp. and *Disporella* sp. produced LMC and HMC in a previous study [49].

The next biggest source of variation was among individuals of the same species, reflecting intraspecific variability. Therefore, biological processes, also known as vital effects [55], seem to be the main factors controlling the skeletal Mg-calcite in these organisms and, in the case of bryozoans, at intra- and interspecific and intracolonial levels [56]. Other studies provide corroborative evidence that the variation in the skeletal Mg-calcite in spirorbid polychaete species [57] and Antarctic bryozoans is partially biologically mediated [24,26].

### Potential environmental influence on skeletal Mg-calcite

Differences among sites (within species) contributed less to variation in skeletal Mg-calcite than among species but was still a significant effect. Several studies have demonstrated that marine calcifiers such as bryozoans, coccoliths, foraminifera and sea stars can respond differently to a range of environmental factors (e.g. water temperature, alkalinity, salinity and Mg/Ca ratio of seawater) [12,24,25,58–60]. The small effect size of differences among sites could reflect the relatively stable environment which occurs in the water column beneath long-lived sea ice. These bays, which are covered by fast sea-ice almost all the year, have little annual variation in seawater temperatures and would therefore have little influence on the skeletal Mg contents in calcite in any species there, as suggested in previous studies focusing on cold water bryozoans [24,25]. However, the differences between sites could be due to variation in other factors such as freshwater input from summer ice melt. Freshwater inputs can affect seawater chemistry and temperature and also transport sediment into nearshore habitats. In Brown Bay for example, freshwater input from a melt stream carries sediment contaminated with metals and hydrocarbons into the bay, which has led to the contamination of sediments [29,37] within the bay and has resulted in effects on benthic communities recruiting to hard surfaces [7]. However, more environmental data from these sites, or a broader range of environmental variation among sites, are required to disentangle the main factors influencing spatial differences in skeletal Mg.

Although depth had the least effect, it was significant, with deeper sites having slightly higher Mg content in calcite, however this result was only due to significantly higher values of Mg content in calcite of *Spirorbis* sp. 2 in deeper waters from three locations (BBOuter, OB2 and OB3). Other species, however, such as *I*. *nutrix* and *Spirorbis* sp.1 exhibited higher values from shallow control locations than deeper samples. Therefore, there was not any clear depth-related pattern. The lack of influence of depth on mineralogy found is probably due to the narrow depth range (10 m) that was sampled.

Although there was no evidence of effects of contaminated sediments on skeletal Mg-calcite concentrations, further studies covering a broader area and focusing on other species, different contaminants or higher contaminant levels are needed to evaluate whether or not the Mg content in calcite might be indirectly influenced by contaminated sediments. Heavy metal toxicity may reduce growth rates of marine invertebrates such as bryozoans and worms [14,15], due to the investment in fitness traits (e.g. growth or reproduction) which are compromised by investment in detoxification mechanisms [61]. Also, organisms with slower growth rates tend to deposit less wt% MgCO_3_ in calcite [5]. Therefore, we could expect lower skeletal Mg content in areas exposed to heavy metals. However, the absence of an effect of contamination could alternatively be due to the fact that these sessile invertebrates are growing on hard surfaces and not in direct contact with the contaminated sediments and thus limiting their exposure.

### Temporal analyses in skeletal Mg-calcite

We document short term temporal analyses in skeletal Mg-calcite (over a 3 year period) for the first time in a human impacted area. Other studies investigated potential temporal variation in skeletal Mg-calcite for greater periods from 30 y to millions of years [26,50]. In our study, temporal variability in skeletal Mg was not detected. Similarly, another study evaluating temporal variability in skeletal Mg-calcite in the Antarctic bryozoan species *Cellaria diversa* Livingstone, 1928 and *Antarcticaetos bubeccata* (Rogick, 1955) over 30 y did not find any significant temporal differences [26]. The lack of temporal variability in skeletal mineralogy means that these species would make excellent long term indicators of the potential effects of environmental change, based on the assumption that such environmental changes (e.g. ocean acidification) would affect skeletal mineralogy.

### Vulnerability of the Antarctic target species in a changing world

Environmental changes could affect survival and calcification of some Antarctic marine calcifiers studied here, particularly those with HMC skeletons and low thermal tolerance [5,9,62]. Spirorbid polychaete tubes are mainly composed of the most vulnerable calcium carbonate minerals to ocean acidification (aragonite, HMC or a mixture of the two) [22]. In our study, *Beania erecta* and *Spirorbis* sp. 1 skeletons contained a high proportion of magnesium ions (with mean wt% MgCO_3_ in calcite (± SD) of 8.1 ± 1.52 and 7.7 ± 0.69, respectively). The solubility of the skeletons of both species is expected to be greater than that of IMC and LMC or even of aragonite [63]. Although some spirorbid polychaetes seem to have the ability to vary their tube mineralogy with seawater chemistry changes, such alterations may result in a deterioration of the tube hardness and elasticity [64]. Consequently, these species are highly likely to be vulnerable to global ocean surface pH reductions of 0.3–0.5 units by the year 2100 [1]. Experimental evidence for this is limited, but the Mediterranean bryozoan *Myriapora truncata* (Pallas, 1766), which has a wt% MgCO_3_ in calcite between 8.0 and 9.5, has been shown to be vulnerable to ocean acidification at a pH of 7.66, with significant loss of skeleton during a short-term experiment [65]. Further work on Antarctic species is required to determine their vulnerability to such predicted changes.

There is an increase of Mg content in cheilostome bryozoans towards lower latitudes, which is attributed to the warmer seawater [50]. Larger values of Mg in two common co-occurring Mediterranean bryozoans *M*. *truncata* and *Pentapora fascialis* (Pallas, 1766) were also found when the colonies were exposed to high temperatures [66]. Spirorbid tubes also show an increase in Mg content with increasing water temperatures [57]. In addition, there are potentially interactive effects of multiple environmental changes (e.g. warming, acidification and pollution) which should be considered as they can co-occur. The combined effects of increased temperature and reduced pH (from increased CO_2_ concentrations) on the widely distributed cheilostome bryozoan *Jellyella tuberculata* (Bosc, 1802), including from subAntarctic regions [67], was found to dissolve their zooids, possibly due to an increase of skeletal Mg content at high temperatures which made the skeletons more susceptible to dissolution under high CO_2_ [68]. Therefore, organisms living in Antarctic coastal areas that have experienced significant warming in recent years (e.g. Western Antarctic Peninsula) could become more vulnerable to environmental changes if their Mg content increases with temperature [69], however it is not known whether this is occurring.

Such changes could potentially lead to shifts in community composition of calcareous marine organisms in future oceans, especially in polar regions [9]. Species with HMC, such as *B*. *erecta* and *Spirorbis* sp. 1, may be particularly vulnerable to the combined effects of ocean acidification and global change, although variation in species-specific responses is anticipated. Potential decreases of some species such as *B*. *erecta*, which is widely distributed and common throughout Antarctica and has colonies which comprise dense mats and may cover large areas of substratum, could indirectly negatively impact other organisms which use their colonies as substrate, food and shelter [34]. This could be expected in colonies living in shallow waters of some Antarctic regions more exposed to increased temperatures such as the Antarctic Peninsula. However, *B*. *erecta*, has a depth range from 0 to >1500 m [67], and cooler deep waters may form refugia for this and other species. Also, *B*. *erecta* is a competitive dominant species, out-competing other bryozoan species like *Inversiula nutrix* and other sessile organisms [70]. Its decrease could favour other less competitive species such as *I*. *nutrix*, which may be more resilient to acidification due to lower Mg content of its skeleton and, consequently, replacing the ecological niche occupied by *B*. *erecta*.

Calcifying marine invertebrates are important components of marine ecosystems and the effects of environmental change at the species level could potentially lead to wider ecosystem level effects. Given the range and variation in mineralogy of calcifying species observed in this study, some species are likely to be more heavily affected than others. Further work is required to better understand the potential impacts of ocean acidification on marine benthic communities, particularly in areas predicted to change rapidly in the near future such as Antarctic shallow waters [71,72]. As rapid reductions in global atmospheric greenhouse gas emissions are unlikely to occur in the near future and a certain amount of climate change and ocean acidification are already assured, it is even more important for polar conservation to manage threats such as pollution that are likely to interact synergistically with climate change, and that are potentially more tractable in the short to medium term [73].
